# Plant networks are more connected by invasive brome and native shrub facilitation in Central California drylands

**DOI:** 10.1038/s41598-024-59868-w

**Published:** 2024-04-18

**Authors:** C. J. Lortie, Charlotte Brown, Stephanie Haas-Desmarais, Jacob Lucero, Ragan Callaway, Jenna Braun, Alessandro Filazzola

**Affiliations:** 1 Department of Biology, York University, Toronto, ON M3J1P3 Canada; 2 Département de Biologie, Université de Sherbrooke, Voie 9, Sherbrooke, Québec J1K 2R1 Canada; 3 Texas A & M, Department of Rangeland, Wildlife and Fisheries Management, 495 Horticulture Rd #305, College Station, TX 77843-2183 USA; 4 Division of Biological Sciences, University of Montana, Missoula, MT 59812 USA; 5 Apex Resource Management Solutions, Ottawa, ON Canada; 6 Department of Biology, University of Western Ontario, London, ON N6A 5B7 Canada

**Keywords:** Annual plant, Community ecology, Facilitation, Foundation species, Invasion, Network analyses, Resilience, Shrubs, Community ecology, Ecology

## Abstract

Dominant vegetation in many ecosystems is an integral component of structure and habitat. In many drylands, native shrubs function as foundation species that benefit other plants and animals. However, invasive exotic plant species can comprise a significant proportion of the vegetation. In Central California drylands, the facilitative shrub *Ephedra californica* and the invasive *Bromus rubens* are widely dispersed and common. Using comprehensive survey data structured by shrub and open gaps for the region, we compared network structure with and without this native shrub canopy and with and without the invasive brome. The presence of the invasive brome profoundly shifted the network measure of centrality in the microsites structured by a shrub canopy (centrality scores increased from 4.3 under shrubs without brome to 6.3, i.e. a relative increase of 42%). This strongly suggests that plant species such as brome can undermine the positive and stabilizing effects of native foundation plant species provided by shrubs in drylands by changing the frequency that the remaining species connect to one another. The net proportion of positive and negative associations was consistent across all microsites (approximately 50% with a total of 14% non-random co-occurrences on average) suggesting that these plant-plant networks are rewired but not more negative. Maintaining resilience in biodiversity thus needs to capitalize on protecting native shrubs whilst also controlling invasive grass species particularly when associated with shrubs.

## Introduction

The movement of species around the globe is a contemporary facet of anthropogenic global change. This introduces exotic species to communities comprised of species that have coexisted for millennia, and some of these exotic species will become invasive^1–3^. Invasive plant species can negatively influence almost every ecological dimension of pattern and process from plant-plant^4,5^, to plant-animal^6,7^, to ecosystem-level functions^8,9^. Declines in resident native diversity, function, and resilience to other exotic species are common negative outcomes^10,11^. As a counterpoint to some of the effects of invasive plant species, foundation native plant species are typically defined as plants that provide significant cover or structure in many systems including drylands^12–14^. This benefactor species can in turn provide structure for and benefits to other plants^15,16^ or animals^17^. Consequently, a deeper understanding of the capacity for these foundation plant species to support diverse and potentially resilient communities regionally is critical^18,19^. Re-establishment of native vegetation is also a common goal in the restoration in many terrestrial habitats^20^. Foundation plant species can thus fulfill both a fundamental and applied purpose supporting the communities they occupy, but the facilitation effects must understood within the broader context of species interactions. Fundamentally, we must ascertain whether native foundation plant species can offset the negative ecological effects of invasive plants^21^ either in terms of preserving a proportion of the native species^22^ or in protecting some of the capacity for interactions between the remaining species locally or regionally to respond to additional changes^23^. Facilitation or positive plant-plant interactions will thus be a pivotal stabilization process provided native foundation species such as shrubs facilitate other natives^24^.

Unfortunately, this is not always the case. Invasive exotic species such as annual plants can often take advantage of these native foundation species^25–27^. Facilitation by native shrubs can become a two-edged sword—as demonstrated in drylands^28^. We need to better understand whether the declines in richness of native plant species in this specific context, whilst clearly negative, are still protected in some capacity through their connections to one another in the remaining associational network^29^. Contrasting the connections between species with and without the effects of foundation species and with and without a highly invasive annual species will highlight the importance of moving beyond single species foci in considering whole-community assembly^30^. Here, we test the hypothesis that the centrality or frequency of associations between resident species within fine-scale ecological networks can be mediated by shrub facilitation in the face of a common, disruptive invasive plant species within a dryland region.

Fine-scale plant ecological surveys provide a powerful lens into local interactions and association patterns. Censuses of diversity, abundance, and habitat structure provided by foundation and dominant plant species are key approaches to infer the importance of microclimate^31^, differences in distribution locally and regionally^32^, richness patterns^33^, and interactions with the environmental drivers of change including soil dynamics^34^. In drylands, i.e. arid and semi-arid grass and mixed shrub lands^35^, a simple two-phase model or categorization is often used to structure vegetation sampling, typically termed shrub/open microsite—but it also can be improved in future research by incorporating distances from shrubs^30^. This structured, simple paired sampling best nonetheless approximates the vegetation mosaic in dryland ecosystems for this region and particularly for sampling across distributed sites^36^. Distributed fine-scale sampling such as paired shrub-open sampling within a region is an effective experimental design for surveys testing for differences in distribution and diversity at scales relevant to plant-plant interactions and invasive species impacts on community assembly in deserts^37,38^. Here, we used this approach to test the generalized hypothesis proposed that a native shrub can mitigate some of the effects of a widespread invasive plant within the central drylands of California using a co-occurrence and interaction network framework to capture salient community dynamics.

## Results

**Figure 1:**
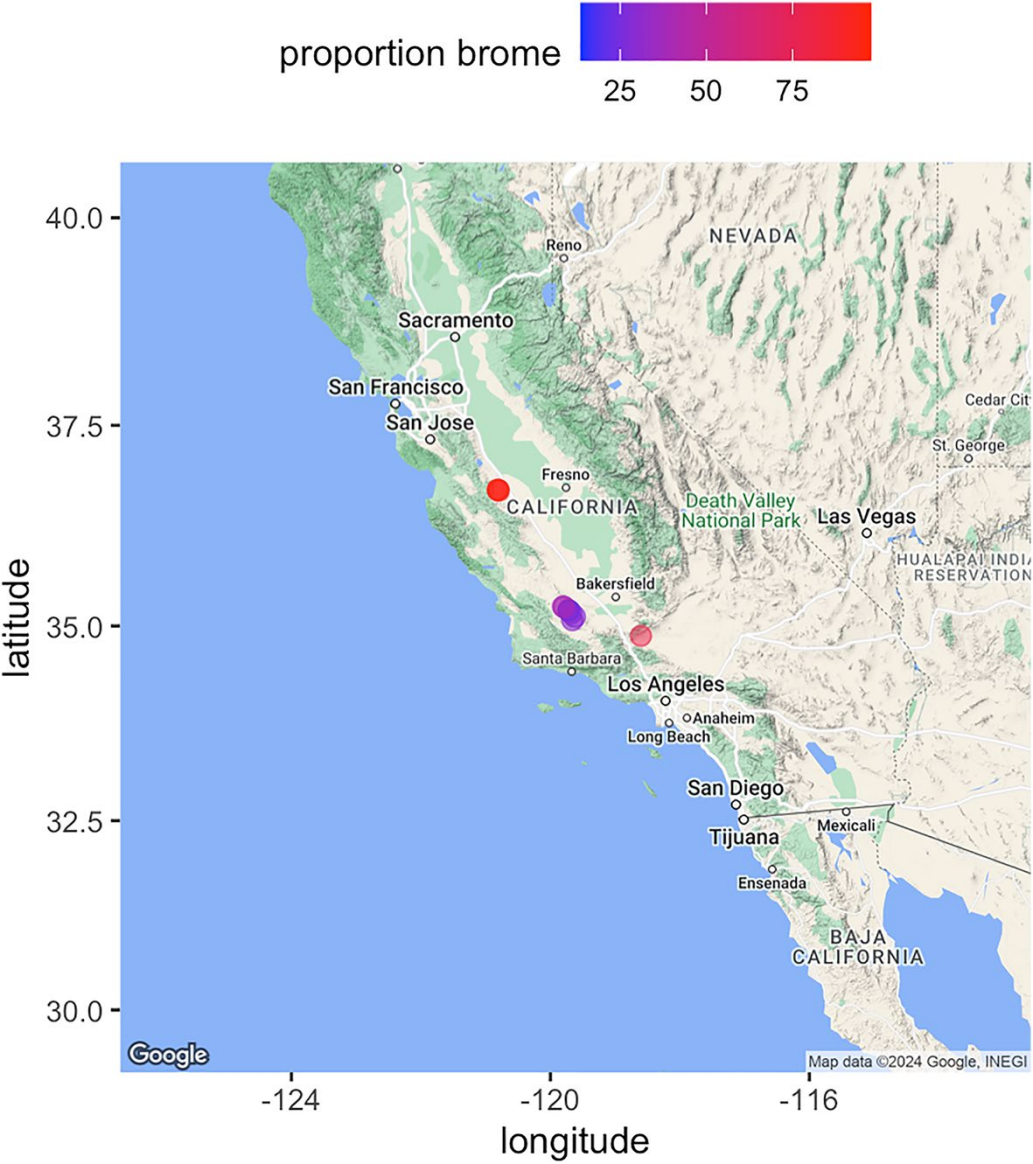
A map of the central drylands of California sampled for patterns in plant associations with and without native shrubs present and with and without a highly invasive plant species. Each point shows a site surveyed for plant occurrences by species. A total of 11 sites were used. Each site was within an arid or semi-arid climate and dominated by the native shrub shrub *Ephedra californica* with the invasive annual plant species *Bromus rubens* present at the site level. The relative mean proportions of total site-level plant abundance comprised by *Bromus rubens* is shown with a heat map per point. The open-source programming language R version 4.3.1 (https://cran.rstudio.com/) was used to create the map. The base layer imagery is sourced from Google (CC BY 4.0, retrieved October 23, 2023) using from the R package ggmap version 3.0.2 (https://cran.r-project.org/web/packages/ggmap/).

In the San Joaquin Desert region^39^, both the native *Ephedra californica* shrub and the invasive *Bromus rubens* are common. *Ephedra californica* is a long-lived shrub reaching heights of 1 m with an extensive canopy^40–42^. It facilitates other species of plants and animals regionally^43–46^. *Bromus rubens* is also common, can reach heights up to 0.5 m^47^, forms a dense, low lying vegetative canopy^48^, and can exclude native plant species in the region^27,49^. Using this two-phase design, we sampled a total of 3196 plots comprising 11,626 individual observations for 54 species across 11 sites within these drylands (Fig. 1, see Methods for full paired shrub-open ecological sampling details). These data are publicly available^50^*. Bromus rubens* was present in 42% of all plots. *Bromus rubens* (i.e. brome) was present under shrubs in 29% of all plots and in the open in 13% of the samples, and brome is thus unfortunately also facilitated alongside native plant species by the foundation species (GLM, Chi-square _microsite_ = − 0.07, *p* = 0.0001). This is consistent with previous research^28^. Brome also reduced the diversity of the natives within the region both under shrubs and in the open (GLM, Chi-square _invaded by brome_ = 1.2, *p* = 0.0001), and this also supports previous research^27,51^. Nonetheless, the fundamental goal here is to ascertain whether its presence influences the associational network of the plant species in the fine-scale context of shrub-open relative to plots with and without the invader.

Ecological network analyses comprise an extensive, evolving set of analytics and theory. The heuristic value of the exhaustive array of ecological network metrics primarily relies on their specificity matched to the available data and in relative differences within a representative, single measure within an ecological region^52–54^. Similar to contrasts for differences in diversity in face of management and theory development, a locally anchored, unified measure within a region is germane to hypothesis testing^55^. Two criteria have been proposed for co-occurrence network analyses, particularly with plants. Availability of regional-scale present-absence data, and previously reported biotic interactions between key species^56^. This study satisfied these criteria. Furthermore, an emerging set of ecological network theory has incorporated ‘keystoneness’ into the global discourse (similar to the term foundation species used in the plant-plant facilitation interaction)^57,58^ and to a lesser extent invasive species effects on networks through metrics associated with centrality^59^. We innovated upon and extended this theory by explicitly using the 2 × 2 ecological contrast described (shrub-open, brome-no brome) and framed the hypothesis testing for centrality. This metric describes the extent that species are functionally keystone and thus more tightly and centrally connected to one another (relative to edge nodes) in each of these specific fine-scale contexts^60^. Here, co-occurrence data were analyzed through this keystone-centrality lens (see Methods for details).

**Figure 2:**
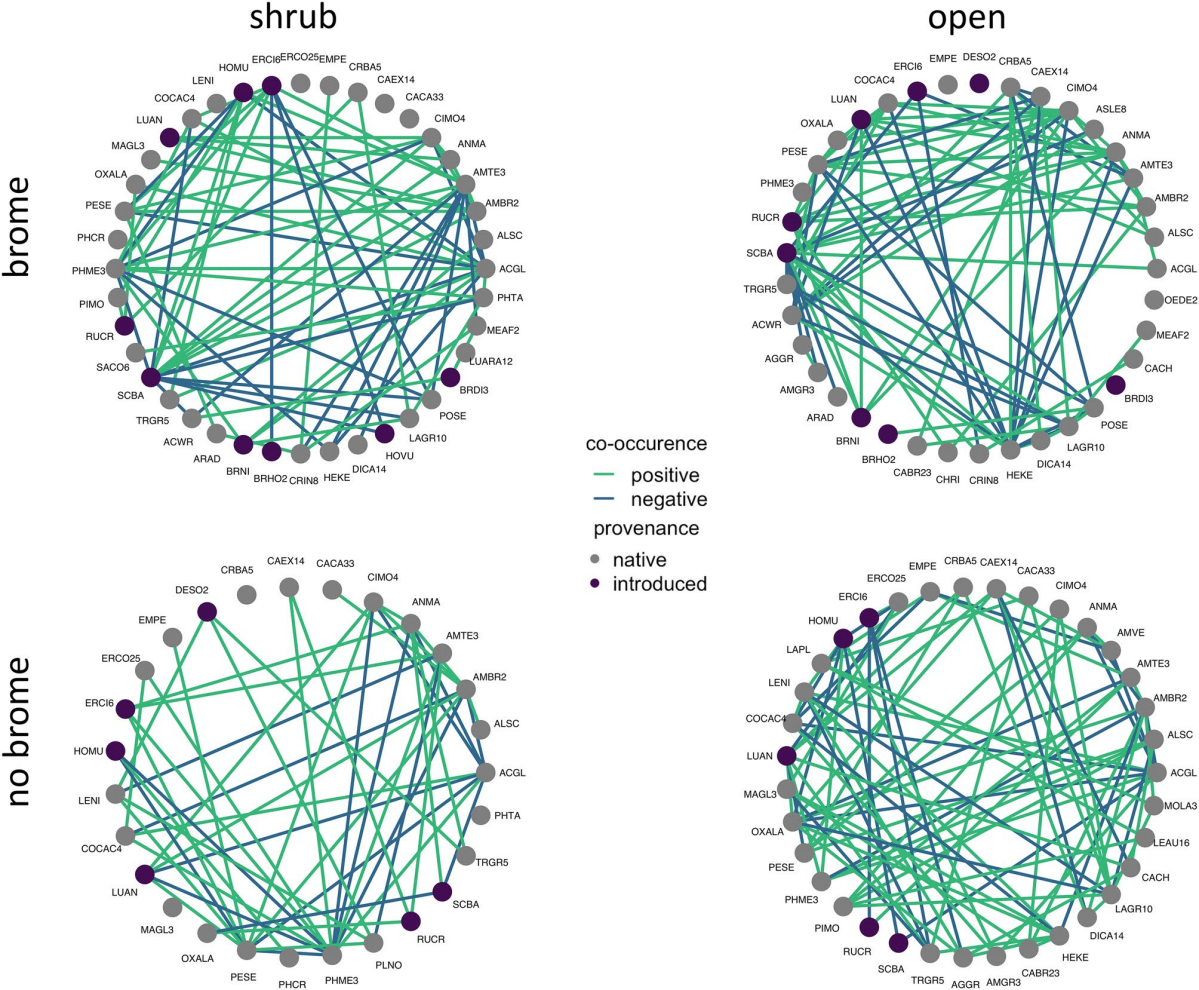
Co-occurrences of species within plant communities in the central drylands of California. Shrub microsites were defined as fine-scale vegetation plots within the canopy of the shrub species *Ephedra californica*, and brome sites were defined as plots with the invasive *Bromus rubens* present. Provenance described the native or introduced status of each species, and co-occurrences were classified as either positively or negatively associated within every pair of species for the respective communities.

Shrub-open, brome-no brome contrasts provided clear insights into the fine-scale dynamics in co-occurrence patterns within the region through this simple vegetation structure as a proxy for microhabitats (Fig. 2). Both native shrubs and brome rewired the connections between resident species from potential non-random associational patterns of species to structured associational co-occurrences delineated by the shrub-open, brome-no brome approach (Table 1, Z-score test for net proportional differences from null, *p* < 0.0002). The lowest frequency of negative species associations was detected under shrubs without brome (Table 1, Z-score test from mean of negative to positive, *p* = 0.005), and whilst brome also influenced association patterns within these fine-scale interaction networks, the net negative-to-positive effect did not differ from the mean regionally (Table 1, Z-score for brome mean net effect, *p* = 0.07). Consequently, the net outcome of the plant-plant interactions between resident plant species is robust from local-to-regional scales at approximately a 50% balance between positive and negative associations (and between 10–15% different totals from non-random co-occurrences). This is profoundly important because it suggests that specific species associations can and will shift with changes in the presence of foundation plant species and an invader, but that at least in the short term, the net outcome for potential competitive and facilitative effects is similar between extant species at the community level^56,61,62^. Invasion does not necessarily shift plant-plant interactions to more negative.

**Table 1:** The relative frequency of associations within plant communities in the central drylands of California.

Microsite	Negative associations	Positive associations	Negative-to-positive ratio	Total non-random
Open, brome	30	55	0.55	12.8
Open, no brome	27	53	0.51	13.4
Shrub, brome	27	47	0.57	9.5
Shrub, no brome	17	34	0.50	14.5

Plant community data sampling was structured using the native shrub *Ephedra californica* with microsites under this species defined as shrub. Paired plots without a shrub canopy were defined as open. Plots were then further categorized as invaded by the invasive annual plant species *Bromus rubens* or as ‘no brome’. The association contrasts provided were the total associations in each group (positive or negative) over the total number of associations for that specific assemblage of species. Total non-random describes the frequencies for a specific association class from data randomization procedures for null models (derived from R the package cooccur).

**Figure 3:**
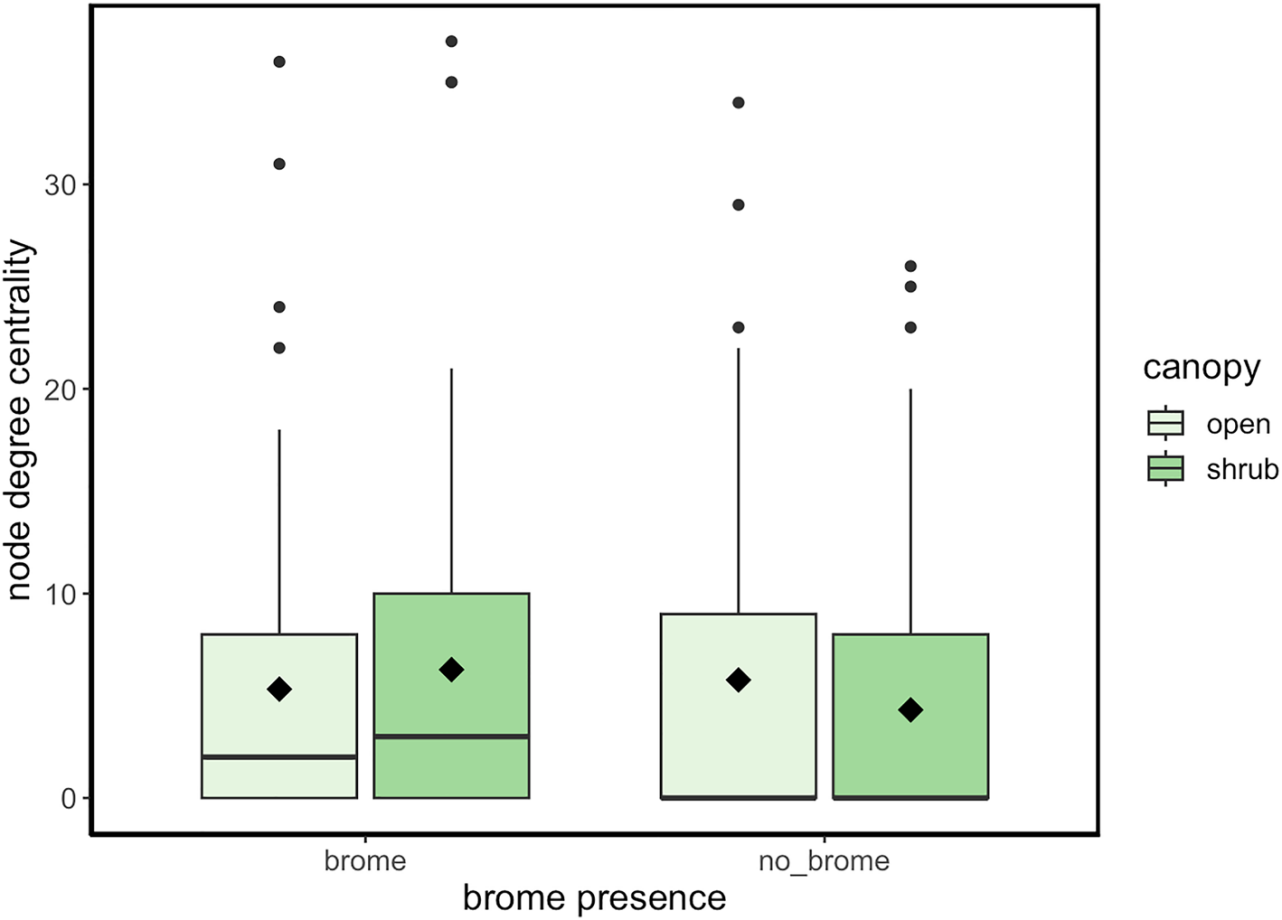
A contrast of the node centrality estimates within plant-plant association networks sampled in the central drylands of California. The comparative box plot show the median value as a central line, lower and upper quartiles bound the box, and the triangles denote the mean value for each grouping level. The canopy plots were sampled under the shrub *Ephedra californica* or in the open without a shrub canopy whilst brome plots were sample instances with the invasive species *Bromus rubens* present in the vegetation. There are no units for the relative metric of node degree centrality.

Despite a largely consistent balance in the net frequency of positive and negative associations between shrub facilitation and invader effects, there are other changes that dryland terrestrial plant communities will face including other novel and likely exotic species and a changing climate^35,59^. Patterns in associational frequency, in addition to sign, between species is also important^63^. Centrality measures significantly differed between shrub and open and with and without brome (Fig. 3, GLMM, Chi-square _microsite x invaded by brome_ = 5.6, *p* = 0.018). Specifically, shrub microsites with brome present had significantly higher relative centrality scores (Fig. 3, GLMM estimated marginal means post hoc contrast, estimate = 0.461, *p* = 0.007). This effect was directly associated with brome under shrubs with a marked increase in centrality from shrubs without brome (i.e., Fig. 3, means of 6.3 relative to 4.3 respectively or a 42% net increase). This difference was not driven by concurrent increases in other exotic species because repeated sensitivity analyses with only natives similarly increased in network centrality with brome and a shrub canopy (GLMM, Chi-square _microsite x invaded by brome_ = 5.7, *p* = 0.02 with estimated marginal means post hoc contrast, estimate = 0.4, *p* = 0.03).

## Discussion

The implication of these robust findings is that individual species under shrubs invaded by brome experience relatively higher degrees of centrality within the networks or assemblages. This implies that these co-occurring species are more highly connected, likely generalist in association, and at least some of the species such as brome can function more fundamentally as keystone species^58^. Higher mean degree centrality is a very simple count of links for each species in each of these specific ecological contexts. This may seem like a positive ecological outcome in terms of resilience, but higher centrality can in some instances reduces resilience and capacity for a set of species to respond to change and disturbance because the species are more frequently associated in space or time^60^. Greater centrality in the network suggests some species are more important, and thus the entire assemblage is sensitive to the loss of one species. For example, in plant-pollinator systems with higher mean degrees of centrality (more frequent associations between individual species), the loss a single species of plant for floral resources or a pollinator species critical for plant reproduction can become important locally if either species is connected to many other species within the community^60^. Species redundancy can offset some of the losses in reliability of key ecological functions, but without experimentation to test for engineering capacities for each species, it is prudent to assume that more flexible networks are a desirable conservation outcome^64^. This same resilience principle is relevant here in plant-plant interaction networks.

More links to each species under shrubs with brome suggests that loss of a species can result in a significant erosion of sets of ecological interactions—whether positive in supporting persistence through amelioration^65^ or negative interactions through indirect competition that can promote higher annual plant diversity in drylands^66^. There was also a significant difference in the contrast between shrub and open microsites without brome, i.e., shrub-only networks were less connected with lower relative centrality (Fig. 3, GLM estimated marginal means post hoc contrast, estimate = 0.366, *p* = 0.37). This further suggests that communities or assemblages of species without brome but facilitated by native shrubs are more resilient and thus able to respond to changes, i.e., fewer specific-species annual plant associations. Less associations per species under shrubs without brome means less to lose per species. Change capacity is supported best through opportunities to shift co-occurrences in annual plant communities^36,67^. Colloquially, all eggs are not in one or fewer baskets. Consequently, the compounded effects of brome in reducing native species diversity coupled with the increasing ‘keystoneness’ under shrubs, that generates more tightly connected species in the remaining communities, suggests that it can usurp the central role of shrubs as agents of positive change in drylands. Brome co-opted the positive effects of native foundation species and established new critical thresholds in diversity and associations that are less robust^10^. Strategies that balance dominant native species conservation and even restoration within these ecosystems must plan for the increased likelihood that sub-dominant exotics will become both particularly invasive into these local ecological opportunities and even undermine responsiveness of the remaining species to future change.

## Conclusions and implications

Interactions with foundation and invasive species are a powerful tool to advance community assembly theory and inform management of habitat through vegetation in dryland ecosystems. Centrality metrics from ecological network theory directly informs species richness given the importance of foundational native and introduced keystone species. It is a strongly complementary measure because it examines composition differences and how they shift in response to invasion (and to the shrubs or keystone species if included in the data structure). This metric provides an alternative, new facet to summarize community structure that is appropriately represented within communities best simplified by foundation and keystone species concepts. It can be used to infer key levers, such as invasion at fine-scales, that can shape plant-plant associations and potential interactions^68^. Based on the present analyses, the restructuring of the plant community is evident. When connections within a community shift with invasion, it is a significant conservation concern. Other plant communities may therefore be vulnerable to the same degree of restructuring observed within this study if a pervasive enough exotic plant becomes established. Although not tested here, the long-term temporal changes that occur within the network can provide useful insights into the potential restructuring over time ^69^. Both the relative proportion and overall density of the invasive species can likely alter the degree of centrality in the network.

Research is needed to examine network stability and resilience in the face of increasing invasions and change. We also need to begin to include other measures of network connectivity and centrality. This is a novel approach, but the findings are nonetheless promising very broadly in invasion biology provided there are clear factors such as distinct microsites or key dominant plants to reasonably anchor analyses. Here, the presence of brome reduced species diversity, but it also had more subtle effects on the plant-plant interactions inferred from the network analysis. There was not a dramatic shift to more negative interactions, but instead, there was a shift to more tightly connected and thus less resilient networks of species^60^. Furthermore, brome increased centrality more under shrubs transforming their capacity as native, foundation species to support diverse and potentially resilient plant communities. Consequently, if native shrubs are to be leveraged for restoration and conservation as viable local sinks for regional biodiversity^70^, targeted control of invasive species such as *Bromus rubens* must selectively and aggressively reduce their presence at these fine-scale hotspots.

## Methods

### Study site and species

The central valley of California comprises an arid and semi-arid climate with vegetation and animals consistent with this climate^39^. It is a mix of diverse land use practices, and all ecological study sites used herein were on open use, protected federal lands. The 11 sites used in this study have been extensively studied and open data are available for key site attributes^51,71^. *Ephedra californica* has been well studied and described in the region as a foundation species for other plants and animals^45,72^. This region is described by many endemic plants and animals^73^. *Bromus madritensis ssp. rubens* is common regionally and highly invasive^74^. Other exotic species that are observed frequently at these sites include *Schismus barbatus*, *Erodium cicutarium*, *Hordeum murinum*. Some of the common native species that occur at these sites include *Amsinckia tessellata*, *Lasthenia gracilis*, and *Leptosyne californica*. The specific compositions of species and respective frequency at each of these sites have been described in previous datasets and studies^36,70,71^, however, at each of these sites, both *E. californica* and *B. rubens* were commons species occupying > 10% of the shrub and annual community respectively. The provenance of observed plant species was derived from the CalFlora database^75^, and the R package taxize was used to harmonize nomenclature and systematics for the province classifications^76^.

### Experimental design

The shrub-open, dyad approach was used to sample vegetation^30^. Random paired-open is a random stratified sampling design for shrub versus open microsite selection. Relative to a fully randomized selection design, this methodology is the most powerful (and common) tool for assessing interactions with foundational plants and their relative ecological effects whilst minimizing potential confounding spatial effects^77^. Quadrat plots were 0.5 × 0.5 m in size. Shrub microsites were sampled by placing these quadrats under the shrub canopy on the north side within the drip line, and open microsites were placed randomly at 1 m away from the paired shrub without any shrub canopy above. Shrubs were randomly selected within the landscape with at least 10 m between each survey pair. We used a quadrat with an area of 0.25 m^2^ because it allows for placement completely within the shrub dripline to capture all species affected by the shrub canopy. Quadrats of this size are also common in other desert shrub facilitation studies (e.g., ^27,36,66^). All plants within the quadrat were surveyed to species at peak flowering, and abundance per species was recorded. A total of 4 years of data from 2015–2018 were sampled at every site consistently at peak flowering in March or April seasonally for a total of 2838 plots. *Post-hoc*, sites with *B. rubens* present were classified as brome or no brome plots for subsequent models. All microsites were selected randomly and without selection bias for brome (or any other species of annual plants). Randomization of sampling was ensured with random number tables for selecting the paired shrub-open microsites within a site.

### Data and statistical models

All data assessment and veracity checks prior to data publication and statistical models were done in the R programming environment version 4.3.0^78^. The tidyverse suite of R packages were used to error check for duplicates and distinct observations^79^. The packages biclust and cooccur were used to establish and calculate associational frequencies between species and the sign of each association (i.e. positive or negative)^80,81^. The base R function prop.test was used to contrast the associational probabilities and generate Z-scores with significance testing weighted by sample sizes^82^. Using an unweighted network analysis^83^, the centrality metric of degree (i.e., a count of the number of node connections relative to edges) was calculated using the package tidygraph^84^. The package glmmTMB and base R GLMs were used to test for differences using generalized linear mixed models^85^. To test if brome was facilitated by shrubs, we fit a GLM model with shrub-open as the predictor. For comparisons of the effects on the entire annual community, we fit a GLM model with both shrub-open and brome-no brome as fixed effects. A negative binomial model was used because the plant abundance data represented discrete values with overdispersion (i.e., the variance exceeded the mean). To test the effects on the network centrality, we fitted mixed models with the same predictors and centrality as the response variable. We used a Gamma distribution in the mixed models because centrality represents a continuous positive value that was right-skewed. The relative importance of sites was explored and plots were treated as random effects to control from natural variation within shrub-open pairs. Annual variation was similarly explored in sensitivity analyses. There were no significant temporal effects on the networks; consequently, relative species occurrences for all years were included and analyzed as a composite set of measures. The total relative abundances per site were also calculated to show the net extent of invasion by brome at each site in the map provided. We explored the relative importance aridity and cover; neither significantly influenced the main findings reported. The package emmeans was used to contrast estimated marginal means from models^86^.

## Data Availability

Plant species survey data are freely available at Figshare. https://figshare.com/articles/dataset/A_survey_the_plant_association_patters_in_the_central_drylands_of_California_USA_/22946267. A list of sites and geolocation data are available at the Knowledge Network for Biocomplexity. https://knb.ecoinformatics.org/view/doi:10.5063/F18914BJ.
